# The beta hairpin structure within ribosomal protein S5 mediates interplay between domains II and IV and regulates HCV IRES function

**DOI:** 10.1093/nar/gkv110

**Published:** 2015-02-24

**Authors:** Prasanna Bhat, Shivaprasad Shwetha, Divya Khandige Sharma, Agnel Praveen Joseph, Narayanaswamy Srinivasan, Saumitra Das

**Affiliations:** 1 Department of Microbiology and Cell Biology, Indian Institute of Science, Bangalore 560 012, India; 2 Molecular Biophysics Unit, Indian Institute of Science, Bangalore 560012, India

## Abstract

Translation initiation in Hepatitis C Virus (HCV) is mediated by Internal Ribosome Entry Site (IRES), which is independent of cap-structure and uses a limited number of canonical initiation factors. During translation initiation IRES–40S complex formation depends on high affinity interaction of IRES with ribosomal proteins. Earlier, it has been shown that ribosomal protein S5 (RPS5) interacts with HCV IRES. Here, we have extensively characterized the HCV IRES–RPS5 interaction and demonstrated its role in IRES function. Computational modelling and RNA–protein interaction studies demonstrated that the beta hairpin structure within RPS5 is critically required for the binding with domains II and IV. Mutations disrupting IRES–RPS5 interaction drastically reduced the 80S complex formation and the corresponding IRES activity. Computational analysis and UV cross-linking experiments using various IRES-mutants revealed interplay between domains II and IV mediated by RPS5. In addition, present study demonstrated that RPS5 interaction is unique to HCV IRES and is not involved in 40S–3′ UTR interaction. Further, partial silencing of RPS5 resulted in preferential inhibition of HCV RNA translation. However, global translation was marginally affected by partial silencing of RPS5. Taken together, results provide novel molecular insights into IRES–RPS5 interaction and unravel its functional significance in mediating internal initiation of translation.

## INTRODUCTION

Hepatitis C Virus (HCV) is a major pathogen which primarily infects the liver. Chronic HCV infection might lead to liver cirrhosis and finally hepatocellular carcinoma (1). Around 170 million people in the world are chronic carriers of HCV (2). HCV is positive sense single stranded RNA virus of the *flaviviridae* family. The genome is organized into a long 5′ untranslated region (UTR), an open reading frame (ORF) and a 3′ UTR. The long ORF codes for a polyprotein which is processed into structural and non-structural proteins (3). Translation of HCV RNA is one of the early steps in the HCV life cycle and is mediated by the Internal Ribosome Entry Site (IRES), which is present in the 5′ UTR of the HCV genome. IRES-mediated translation initiation in HCV is independent of the cap-structure and uses only a small subset of canonical initiation factors (4). During translation initiation, the 40S ribosomal subunit binds to the HCV IRES to form a IRES–40S complex and this promotes the recruitment of eIF3 and eIF2/GTP/Met-tRNAMeti to form the 48S complex (4). Subsequent guanosine triphosphate (GTP) hydrolysis, release of eIFs and recruitment of the 60S subunit lead to formation of the 80S ribosome complex (4). Thus, the translation initiation mechanism in HCV is quite different from canonical cap-dependent translation (4–6).

As only a subset of canonical initiation factors are involved, RNA elements present in the HCV IRES play a major role in the internal initiation of translation (4). HCV IRES is organized into three domains, namely domains II, III and IV. Domain II is necessary for proper conformational change in 40S during IRES–40S complex formation (7). In addition, domain II is also necessary for eIF2 release and 80S formation from the 48S complex (8). Domain III is critical for 40S recruitment and IRES–eIF3 interactions (4). Domain IV contains the initiator AUG and the integrity of domain IV is essential for IRES activity (9). The pseudo knot structure is shown to be involved in proper positioning of 40S at the initiator AUG (10). Recruitment of 40S and formation of a functional 40S–IRES complex depends on high affinity interactions between RNA elements in the IRES and the ribosomal proteins and rRNA (11–15). From literature it is known that ribosomal proteins S2, S3, S3a, S5, S10, S14, S15, S16/S18, S27 and p40 interact with the HCV IRES (11–16). In particular, the interaction of ribosomal protein S5 with HCV IRES has been identified by UV cross-linking of 40S subunit with HCV IRES. Interestingly RPS5 was the only ribosomal protein that cross-linked with the HCV IRES upon UV irradiation (14,15). Earlier studies have shown that RPS5 binding to the HCV IRES can be prevented by a short RNA derived from the IRES and this also inhibits ribosome assembly on the IRES (17,18). Cryo-electron microscopy (cryo-EM) studies of HCV IRES bound to the 40S subunit and the 80S ribosome have provided a general idea of the positioning of the IRES on the ribosome (7,19). Cryo-EM studies have also mapped RPS5 close to the apex of domain II of the HCV IRES (7,19). Although cryo-EM studies on CSFV IRES bound to the 40S subunit provided greater details about interactions involved in HCV-like IRES and 40S complex formation (20), in depth information on HCV IRES–RPS5 interactions was lacking. The exact regions of RPS5 and HCV IRES that are involved in the interactions and their precise role in ribosome assembly have not been characterized. The HCV 3′ UTR is also reported to interact with the 40S subunit and affect HCV translation (21), but it is not known whether RPS5 is involved in this interaction.

In the present study, we have characterized the RPS5–IRES interaction using computational modelling and mutational analyses. Peptides derived from the predicted RNA binding region of RPS5 and mutant RPS5 proteins were used in RNA–protein interaction studies to identify the region of RPS5 involved in HCV IRES–RPS5 interactions. Also, we have extended our study to understand the structure and sequence of RNA elements in the HCV IRES that are important for IRES–RPS5 interaction using IRES mutants. Further, the role of IRES–RPS5 interaction in ribosome assembly on the HCV IRES has been studied using a range of IRES mutants. Finally, using dual approaches, by partial knockdown of RPS5 and *in vitro* translation in presence of anti-RPS5 antibody, we have demonstrated the critical requirement of RPS5 in HCV IRES function. Results provided novel insights into HCV IRES–RPS5 interaction and possible role of this interaction in translation initiation.

## MATERIALS AND METHODS

### Cells, plasmids and peptides

Huh7, Huh7.5 and Huh7 cells harbouring HCV subgenomic replicon (Rep2a) cells were grown in Dulbecco's modified Eagle's medium (DMEM) (Sigma) supplemented with 10% fetal bovine serum. Rep2a cells (a kind gift from Prof. Ralf Bartenschlager, Heidelberg University) were maintained in the presence of 25 mg/ml of hygromycin B. pET-28a(+)RPS5-WT was generated by cloning wild-type (WT) RPS5 in pET-28a(+) vector between BamHI and HindIII sites (a generous gift from S. ShuetsuFukushi, BioMedical Laboratories, Saitama, Japan). pET-28a (+)rRPS5-βbhp was generated by inverse polymerase chain reaction (PCR), using a single pair of partially complementary primers (22). pCD HCV Bicis construct contains Renilla luciferase reporter (RLuc) in the upstream region and Firefly luciferase reporter (FLuc) downstream to HCV IRES (17). All the IRES mutant HCV bicistronic constructs were generated by inverse PCR, using a single pair of partially complementary primers (22). pCD HCV 3′ UTR construct was used to transcribe HCV 3′ UTR RNA. S5M1, S5C2 and NSPS5 peptides were custom synthesized from Genemed Synthesis Inc. Anti-RPS5 mouse monoclonal antibody (Abcam) was used to detect RPS5 protein in western blot analysis. Anti-RPS5 polyclonal antibody generated in our laboratory by injecting full-length recombinant RPS5 antigen into rabbit and affinity purified was used for *in vitro* trasnslation assay to block RPS5. siRPS5 (siGENOMESMARTpool) and siNSP were obtained from Dharmacon. siGENOMESMARTpool contains four different siRNAs targeting same mRNA.

siRNA D-010498-05, RPS5:GAACUCCUAUGCCAUUAAG

siRNA D-010498-04, RPS5:GCACCGAUGAUGUGCAGAU

siRNA D-010498-03, RPS5:CCGCCAAACGCUUCCGCAA

siRNA D-010498-02, RPS5:ACAUUUCCCUGCAGGAUUA

### Computational model of S5–HCV IRES interactions using cryo-EM data as a framework

#### Cryo-EM model as a framework to identify binding components and orientation in IRES–RPS5 complex

Atomic model of eukaryotic 40S ribosome (23) was fitted into the low resolution cryo-EM derived map of HCV IRES–80S complex (19) to identify interacting surfaces involving RPS5 and IRES domains (Supplementary Figure S1A). To account for relative conformational change in the ribosomal head, the 40S ribosomal head was selectively optimized to fit into the density by prior selection of components using chimera and the correlation (about zero) scores are calculated for the fits. In addition NMR model of domain II (24) (PDB ID: 1P5O) was fitted in the IRES density at the Exit site (19) to identify interacting surfaces involving RPS5 and IRES domains. To identify the nucleotides and amino acids involved in the HCV IRES–RPS5 interaction, docking of RPS5 and IRES domains was performed keeping residues found at the interaction surfaces as Ambiguous Interaction Restraints (Supplementary Figure S1B). RPS5 structure used in the docking studies was generated using Modeller v9.2 (25) with the eukaryotic ribosome crystal structure (26) as template (Supplementary Figure S1C).

#### Docking RPS5 and domain II

HADDOCK was used for obtaining an energetically favourable pose of interaction between RPS5 and domain II (27). Surface exposed residues occurring at the cryo-EM based model interface were chosen to guide the docking. Distance constraint of 5 ± 3 Å was used to restrict the interacting segments in the orientation observed in the cryo-EM-based model. For RPS5, residues from the two regions involving the C-terminal 40 residue stretch (Leu103, Arg164, Asn165 and Lys167) and the beta-hairpin (Arg127, Gly132 and Thr133), were considered for applying the constraint. On the other hand, the bases from the sets (A70, G71, G100 and U101) and (C83, C84, A85, U86 and U90) were used as complementary interaction partners. From each of the two interaction regions, one or two residue–base distance constraints were applied at a time and several docking runs were carried out with different combinations. The distances were calculated between C-β or C-γ atoms of amino acid residues and mainly phosphate or sometimes C1′or N1 atoms of RNA bases. Among the docked poses, the one with the least energy and having binding orientation similar to the cryo-EM derived model was selected.

#### Model of domain IV and RPS5 interaction

To determine the potential amino acids and nucleotides involved in the domain IV and RPS5 interaction, we modelled the domain IV region of IRES as a single stranded RNA and positioned the start codon at the P-site using the known mRNA and tRNA bound ribosome structures as template (28) (Supplementary Figure S1D). Mode RNA was used to perform modelling of RNA based on a template structure (29)

### *In vitro* transcription

*In vitro* transcription was performed using T7 RNA polymerase (Fermentas) as recommended by the manufacturer. Wild type and mutant HCV IRES transcripts (18–383nt) were synthesized using PCR amplicon as template which was obtained with T7 promoter sequence fused forward primer and HCV383 reverse primer. α-^32^P body labelled RNA was synthesized using same template in the presence of α-^32^PUTP.HCV bicistronic RNA was synthesized by *in vitro* run-off transcription using pCD HCV bicistronic DNA digested with PmeI (17). HCV 3′ UTR RNA was transcribed using PCR amplicon fused with T7 promoter sequence as template. T7 promoter sequenced fused amplicon was obtained by performing PCR with T7 forward primer and 3′ UTR reverse primer using pCDHCV 3′ UTR DNA construct as template.

### Filter binding assay

Filter binding assay was performed as described earlier (17). The α-^32^P-labelled HCV 18–383 RNA was incubated with increasing concentrations of peptides at 30°C for 15 min in RNA binding buffer (5 mM 4-(2-hydroxyethyl)-1-piperazineethanesulfonic acid (HEPES), pH 7.6, 25 mMKCl, 2 mM MgCl_2_, 3.8% glycerol, 2 mM dithiothreitol and 0.1 mM ethylenediaminetetraacetic acid (EDTA)), and loaded onto nitrocellulose filters. The filters were then washed twice with 1 ml of ice-cold 1× binding buffer and once with absolute ethanol dried. The counts retained on the membranes were measured with a liquid scintillation counter. *K*_d_ values were calculated using nonlinear regression applied by GraphPad Prism 6. Each experiment was repeated three times.

### UV cross-linking assay

UV cross-linking assay was performed as reported earlier (17). In UV cross linking assay α-^32^P-labelled HCV IRES RNA (18–383) or HCV 3′ UTR was allowed to form complex with the purified recombinant ribosomal protein S5 (rRPS5) or purified 40S ribosome subunit in binding buffer (5 mM HEPES pH 7.6, 25 mM KCl, 2 mM MgCl_2_, 3.8% glycerol, 2 mM dithiothreitol, 0.1 mM EDTA) at 30°C for 15 min. These RNA–protein complexes were then irradiated with UV light (254 nm) for 25 min on ice. The excess RNA was digested with RNase A (Sigma) for 30 min at 37°C and UV cross-linked complex was resolved on SDS-polyacrylamide gel, which was dried before it was exposed to the phosphor-Imager cassette. In case of competition UV cross-linking experiments, RNA–protein complex formation was performed in presence of the un-labelled RNAs or peptides.

### Purification of wild type and mutant RPS5

Human ribosomal protein S5 was expressed in BL21 DE3 and purified as mentioned earlier (17). In brief, *Escherichia coli* BL21 DE3 cells were transformed with the plasmid pET-28(a)-WTRPS5 or pET-28(a)RPS5-βhpt expressing the N terminally poly(His)‐tagged wild type or mutant human RPS5 protein. Protein expression was induced by 0.4 mM Isopropyl β-D-1-thiogalactopyranoside (IPTG) and incubated at 16°C for 22 h. Later protein was purified using Ni^2+^–nitrilotriacetic acid–agarose (Qiagen, Hilden, Germany) under non‐denaturing conditions and eluted with elution buffer containing 250 and 500 mM imidazole.

### Purification of 40S ribosome subunits

40S subunits were isolated from Huh7 cells. In brief the cells were suspended in 4 volumes of buffer (20 mM Tris–HC1 pH 7.5, 100 mM KCl, 5 mM MgCl_2_, 5 mM 2-mercaptoethanol) containing 0.5% of non-ionic detergent NP-40 and homogenized on ice using a Dounce homogenizer. Lysate was centrifuged for 15 min at 30 000g, 4°C to remove cell debris. 40S subunits were isolated from the cytoplasmic extract as described earlier (30).

### *In vitro* translation assay

*In vitro* translation assay of the wild type and mutant-capped HCV bicistronic mRNAs were carried out as performed earlier (17). One microgram of bicistronic RNA was used per reaction. The reaction mixtures were incubated at 30°C for 90 min and assayed for both Renilla and Firefly luciferase activity using dual lucifearse kit (Promega).

### Ribosomal assembly assay

^32^P-labelled wild type or mutant HCV IRES RNA (3 × 10^5^ c.p.m.) was added to 25 μl of translation reaction containing 17.5 μl of rabbit reticulocyte lysate (RRL). The reactions were incubated at 30°C for 15 min and diluted to 150 μl with ice cold gradient buffer (20 mM Tris–HCl pH 7.5, 100 mM KCl, 3 mM MgCl_2_, 1 mM Dithiothreitol (DTT)) and overlaid in 5–30% sucrose gradient. The ribosomal complexes were separated by ultracentrifuge for 3 h at 30 000 rpm at 4°C in TH-641 rotor (Thermo Scientific). Fractions (330 μl) were manually collected from the top of the tube and radioactivity was measured in a liquid scintillation counter. To validate the peaks, *in vitro* translation was performed in presence of 2 mM 5′-guanylyl imidodiphosphate (GMP-PNP) or 20 mM of cycloheximide and peaks were resolved on sucrose gradient.

### Transfection

Rep2a or Huh7 cells were seeded into 6-well plates or 12-well plates and transfected with siRNA 16 h after seeding. Transfection was performed in serum and antibiotic free medium (OPTIMEM) using Lipofectamine 2000 (Invitrogen). In case of Rep2a, cells were harvested 72 h post-transfection. To study the role of RPS5 on cap dependent translation Huh7 cells transfected with RPS5 siRNA and after 48 h, the cells were again transfected with HCV bicistronic RNA. These cells were harvested 10 h post-transfection of HCV bicistronic RNA.

### Western blot

Cells were harvested and lysed in 1× RIPA buffer. One hundred micrograms of protein was resolved using SDS-PAGE and transferred to nitrocellulose membrane by semi dry transfer apparatus (Bio-Rad). RPS5 was probed by using anti-RPS5 monoclonal antibody (1:2000 dilutions in 2% Bovine serum albumin (BSA) in Tris Buffered Saline (TBS)) followed by secondary anti-mouse antibody conjugated to HRP (Sigma). Actin was used as loading control. Actin was detected using anti-actin monoclonal antibody conjugated to horseradish peroxidase (Sigma).

### ^35^S-Methionine labelling

Huh7 cells were seeded into 6-well plates and transfected with either non-specific siRNA (siNSP) or siRNA directed against RPS5 (siRPS5). Forty-eight hours after transfection, medium was replaced with serum free DMEM lacking methionine and cysteine (Sigma) and incubated for 1 h. Then 100 μci of ^35^S *in vivo* pro-twin label mix (BRIT) was added to each well and cells were labelled for 30 min. Cells treated with 100 μg/ml concentration of cycloheximide were taken as positive control for global translation shut down. After labelling the cells, media was removed and the cells were washed three times with ice-cold phosphate buffered saline (PBS). Cells were harvested and lysed in the 100 μl of RIPA buffer lacking SDS (50 mM Tris–HCl pH 7.4, 150 mM NaCl, 1 mM EDTA, 1% NP-40, 0.25% sodiumdeoxycholate) containing protease inhibitors (complete EDTA-free protease inhibitor, Roche). The protein concentration of the lysate was then estimated by Bradford assay. Twenty-five micrograms of protein from each sample was mixed with 0.1 mg BSA and ice-cold TCA was added to a final concentration of 20%. The samples were incubated for 30 min on ice and loaded on prewet (with 20% TCA) glass microfibre filters (Whatmann). The filters were washed with 1 ml of 20% TCA twice and once with 1 ml of 100% ethanol. The filters were then incubated overnight with 4 ml of scintillation liquid and counts were taken.

### Polysome profiling

Huh7 cells were seeded in 60 mm dishes and transfected with siNSP or siRPS5. After 48 h, cells were treated with 100 μg/ml of cycloheximide for 10 min at 37°C. Cells were washed once with ice cold PBS-containing cycloheximide and hypotonic buffer (5 mM Tris–HCl pH 7.5, 1.5 mM KCl, 5 mM MgCl_2_, 100 μg/ml cycloheximide). Cells were harvested in ice-cold lysis buffer (5 mM Tris–HCl pH 7.5, 1.5 mM KCl, 5 mM MgCl_2_, 100 μg/ml cycloheximide, 1 mM DTT, 200 U/ml RNAsein, 200 μg/ml tRNA, 0.5% Triton-X-100, 0.5% sodium deoxycholate, 1× protease inhibitor cocktail), incubated on ice for 15 min and KCl concentration in the lysate was adjusted to 150 mM. Cells were centrifuged at 3000g for 8 min at 4°C. Supernatant was processed immediately or flash frozen and stored at −70°C for later use. Lysate equivalent of 200 μg RNA was loaded on to a 15–50% sucrose gradient and centrifuged at 36 000 rpm for 2 h at 4°C in SW41 rotor (Beckman). We used the Density Gradient Fractionation System (ISCO) to fractionate the gradients at a flow rate of 0.75 ml/min with the UV-detector sensitivity set at 1.0. To study the level of 40S and 60S subunits lysate treated with 100 mM EDTA was loaded on 5–30% sucrose gradient containing 25 mM EDTA and centrifuged at 38 000 rpm for 3.5 h at 4°C in SW41 rotor (Beckman) as described by Huang *et al*. (31).

## RESULTS

### Beta hairpin structure present in RPS5 is important for IRES–RPS5 interaction

**Figure 1.**
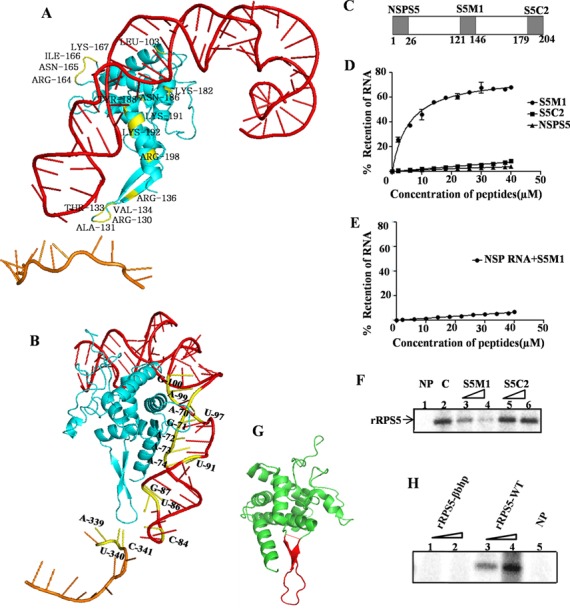
Characterization of the region of RPS5 involved in HCV IRES–RPS5 interaction. (**A**) Model depicting the interaction of RPS5 with the domains of IRES. The amino acid residues of RPS5 involved in the interaction are indicated in yellow, the RPS5 protein in blue, the domain II in red and the SLIV in brown. (**B**) Model depicting interaction of RPS5 with the domains of IRES with the nucleotides involved in interaction indicated in yellow. (**C**) Schematic representation of peptides derived from RNA binding region of RPS5. (**D**) Filter binding analysis of the interaction between HCV IRES and peptides derived from RNA binding region of RPS5 protein (S5M1 and S5C2) and a non-specific peptide (NSPS5). The indicated peptides were incubated with 25fmol of ^32^P body labelled HCV IRES at 30°C before applying it on nitrocellulose membrane. Percentage of RNA retained on the nitrocellulose membrane was plotted against peptide concentration. Average of three independent experiments was used to plot the graph. Apparent *K*_d_ values were calculated using nonlinear regression applied by Graphpad Prism 6. (**E**) Filter binding analysis of the interaction between non-specific RNA (NSP RNA) and S5M1. Average of three independent experiments was used to plot the graph. (**F**) Competition UV cross-linking of HCV IRES and RPS5 in the presence of S5 derived peptides. This is a representative image of three independent experiments. ^32^P-labelled HCV IRES (100 fmol) was incubated with 4 pmol of rRPS5 protein in presence of 100- and 200-fold molar excess of S5M1 or S5C2 peptides. ‘C’ refers to the control lane with no competing peptide.NP refers to the no protein lane. (**G**) The structure of S5. The stretch of amino acids containing beta hairpin structure is highlighted in red. (**H**) UV cross-linking of HCV IRES (100 fmol) with increasing concentrations (2 and 4 pmol) of either wild-type rRPS5(rRPS5-WT) or the beta hairpin deletion mutant of RPS5 (rRPS5-βbhp).

The HCV IRES interacts with a number of 40S ribosomal proteins to form the IRES–40S subunit complex. Recently through computational modelling and fitting in of the cryo-EM density map, we identified the regions in the IRES as well as the ribosomal proteins that are important for this interaction (32). One such protein was the ribosomal protein S5 and our present study is focussed on characterizing the interaction between RPS5 and the HCV IRES. For this purpose, the structure of the HCV IRES–80S complex solved at 15 Å resolution (19) was used as a framework to identify the interactions involving IRES and RPS5. Alignment of the cryo-EM derived structure of the human ribosome (23) with the HCV IRES–80S complex indicated putative surfaces of contact of RPS5 with HCV IRES. The 40S ribosomal head fits in the density with a correlation coefficient of 0.69. The NMR model of IRES domain II was fitted in the low resolution density of IRES–80S complex (19). Further, docking of domain II and RPS5 was performed to identify the nucleotides and amino acids involved in domain II–RPS5 interaction. Keeping residues found at the interaction surfaces as Ambiguous Interaction Restraints, docking of domain II and RPS5 was performed using HADDOCK (27) to obtain an energetically favourable pose of interaction between RPS5 and domain II. The docking study suggested potential contact points between RPS5 and domain II (Figure 1A and 1B, Supplementary Table 1). The RPS5 model used for docking was obtained by homology modelling. From docking studies we noted that the C-terminal region and beta-hairpin of RPS5 interact with domain II. This model for interaction between RPS5 and domain II is in agreement with the recent work of Filbin *et al*. (33). In parallel, mRNA and tRNA bound ribosome structure was used as template to study the interaction of domain IV and RPS5 (28). We modelled the SLIV region of IRES as a single stranded RNA and positioned the start codon at the P-site using the known mRNA and tRNA bound ribosome structures as templates (28). This homology modelling study suggested that in addition to domain II, RPS5 makes interaction with domain IV as well (Figures 1A and 1B, Supplementary Table 1). Taken together, the computational model suggests that ribosomal protein S5 makes multiple contacts with domains II and IV of HCV IRES.

**Figure 2.**
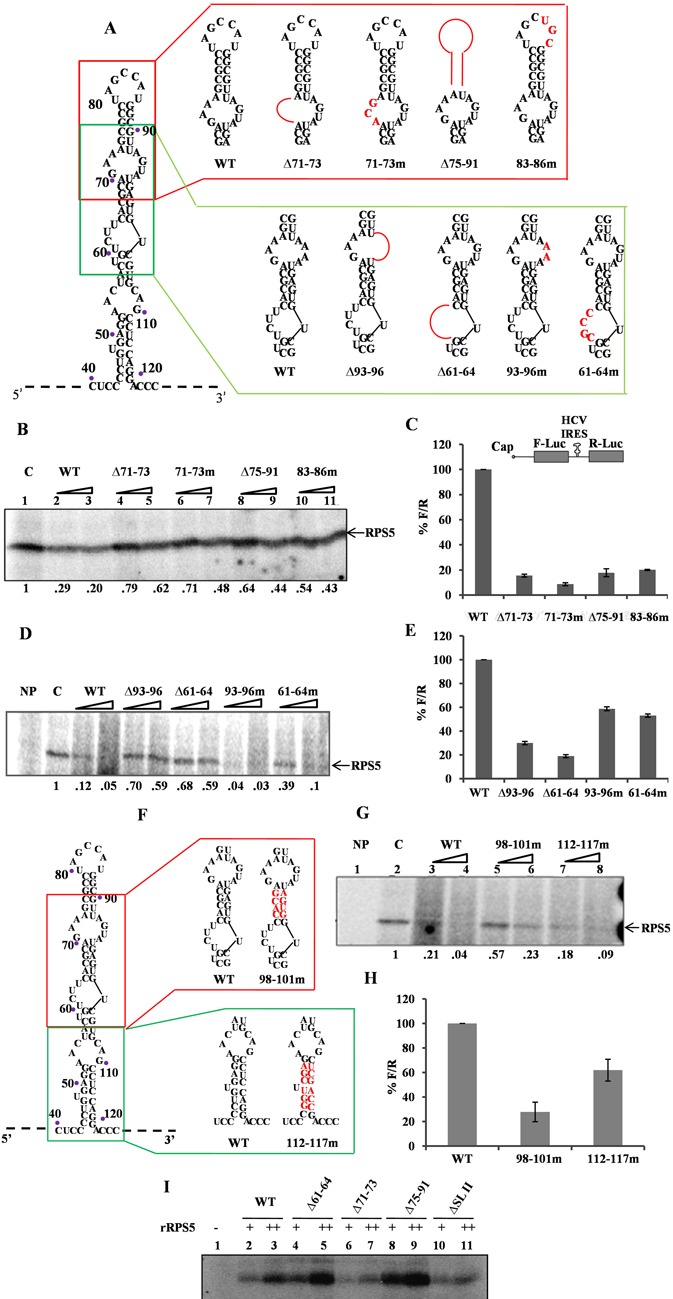
Characterization of RNA elements within HCV IRES domain II important for HCV IRES–RPS5 interaction. (**A**) Schematic representation of HCV IRES domain II wild type and mutant constructs used in the study. Mutated regions are shown in red. (**B** and **D**) Competition UV cross-linking of 40S ribosomal subunit with wild-type HCV IRES in presence of either unlabelled wild-type HCV IRES or domain II mutant IRES RNAs. This is a representative image of three independent experiments. (**C** and **E**) *In vitro* translation of wild type and mutant HCV bicistronic RNAs using rabbit reticulocyte lysate (RRL). Graph represents percentage luciferase activity (% F/R) for wild type and mutant bicistronic RNAs. Graph represents the average of three independent experiments. (**F**) Schematic representation of HCV IRES domain II wild type and mutant constructs used in the study. Mutated regions are shown in red. (**G**) Competition UV cross-linking of 40S ribosomal subunit with wild type HCV IRES in presence of either unlabelled wild type HCV IRES or domain II mutant IRES RNAs. This is a representative image of three independent experiments. (**H**) *In vitro* translation of wild type and mutant HCV bicistronic RNAs using rabbit reticulocyte lysate (RRL). Graph represents the average of three independent experiments. (**I**) UV cross-linking of ^32^P wild type and mutant HCV IRES RNAs (100 pmol) with increasing concentration (2 and 4 pmol) of recombinant RPS5. This is a representative image of three independent experiments.

Next, we aimed to validate the results obtained by computational modelling through other experimental approaches. We started with the regions in RPS5 that were predicted to interact with the HCV IRES and designed two synthetic 26aa long peptides from these predicted regions, namely S5M1 (P121-R146) and S5C2 (N179-R204) (Figure 1C). S5M1 peptide contains a stretch of amino acids that forms a beta hairpin structure in RPS5 and S5C2 peptide was derived from the C-terminal end of RPS5. The interaction of these peptides with HCV IRES was studied by filter binding assays where 25 fmol of ^32^P-labelled HCV IRES was incubated with increasing concentrations of the respective peptides and applied onto nitrocellulose membranes. The count from peptide bound RNA retained on the membrane after suction was taken using a liquid scintillation counter. Percentage of RNA bound was plotted against the peptide concentration. The dissociation constant *K*_d_ was determined using GraphPad Prism 6 software. The S5M1 peptide (*K*_d_ = 5.60 ± 0.67μM) showed specific interaction with the HCV IRES but not with a non-specific RNA (NSP RNA) (Figure 1D and E). The S5C2 and non-specific peptides (NSPS5 peptide) did not show any appreciable interaction with the HCV IRES (Figure 1D). Since S5M1 is derived from RPS5, we performed competition UV cross-linking experiments to check whether both S5M1 and the recombinant RPS5 protein bind to the same region in HCV IRES (Figure 1F). ^32^P-labelled HCV IRES (100 fmol) was cross-linked with 4 pmol of recombinant RPS5 (rRPS5) in the presence of 100- and 200-fold molar excess of either the S5M1 or S5C2 peptide. S5M1 peptide competed with full-length rRPS5 for binding with the HCV IRES while S5C2 peptide could not interfere with IRES-RPS5. Since S5M1 peptide showed specific interaction with HCV IRES and contains an amino acid stretch forming a beta hairpin, we deleted the stretch of amino acids (E123-D140) from RPS5 corresponding to this beta hairpin and replaced it with a tetra glycine bridge to maintain the integrity of the protein structure (Figure 1G). Wild-type rRPS5 (rRPS5-WT) and rRPS5 beta hairpin mutant (rRPS5-βhp) proteins were expressed and purified from bacteria. UV cross-linking of ^32^P-labelled HCV IRES (100 fmol) with increasing concentration (2 and 4 pmol) of rRPS5-WTand rRPS5-βhp proteins was performed. Deletion of beta hairpin in RPS5 completely abrogated its interaction with HCV IRES which further supports for a role of the beta hairpin in IRES–RPS5 interaction (Figure 1H).

### RNA elements in domain II play important role in IRES–RPS5 interaction and ribosome assembly

To study the RNA elements of domain II which are important for IRES-RPS5 interaction, we generated many deletion and substitution mutations in the context of full-length IRES based on the computational modelling results (Figure 2A). We have focussed on specific loops and stem regions containing the predicted nucleotides instead of single nucleotide mutations. We performed competition UV cross-linking assays to study the effect of these mutations on IRES–RPS5 interaction. From literature it is well known that RPS5 is the only 40S ribosomal protein which gets cross-linked with HCV IRES by UV irradiation (14,15). So, in the present study, we have used purified 40S ribosomal subunits as a source for RPS5 protein. ^32^P-labelled HCV wild-type IRES (100 fmol) was UV cross-linked with 40S ribosomal subunits (5 pmol) purified from Huh7 cells, in the presence of either unlabelled wild-type RNA or mutant IRES RNA (100- and 200-fold molar excess). RNA–protein complexes were separated on SDS-PAGE and analysed by phosphor imaging. Since, it is a competition UV cross-linking assay, reduction in band intensity directly correlates with the binding affinity of IRES RNA for RPS5. First, we generated deletions (Δ71–73, Δ75–91) and substitution mutations (71–73m, 83–86m) in the loop regions of domain II that were predicted to interact with RPS5 (Figure 2A). All these mutants showed compromised interaction with RPS5 in competition UV cross-linking assay (Figure 2B). In parallel, we also studied the effect of these mutations on HCV IRES activity by *in vitro* translation in Rabbit Reticulocyte Lysate (RRL) using HCV bicistronic RNA-containing respective mutations and all the four mutants showed reduced IRES activity (Figure 2C).

Next, we studied the role of loop regions in the IRES that are present neighbouring to the predicted RPS5 interacting regions, through deletions (Δ93–96, Δ61–64) or substitution mutations (93–96m, 61–64m) (Figure 2A). To investigate the effect of these mutations on HCV IRES–RPS5 interaction we performed competition UV cross-linking assay. In this assay, ^32^P-labelled HCV wild-type IRES (100 fmol) was UV cross-linked with 40S ribosomal subunits (5 pmol) purified from Huh7 cells, in the presence of either unlabellled wild-type RNA or mutant IRES RNAs (100- and 200-fold molar excess). RNA–protein complexes were separated on SDS-PAGE and analysed by phosphor imaging. Competition UV cross-linking assays of these mutants with 40S ribosomal subunit suggested that Δ93–96, Δ61–64 deletion mutants bind poorly with RPS5, whereas 61–64m showed moderately reduced interaction with RPS5 and interaction of 93–96m mutant with RPS5 was not significantly affected (Figure 2D). From *in vitro* translation experiments, we found that IRES activity was significantly reduced in case of Δ93–96, Δ61–64 mutants and moderately affected in case of 93–96m, 61–64m mutants (Figure 2E).

Further to study the role of nucleotides in the stem regions of domain II that are predicted to interact with RPS5, we generated substitution mutations (98–101m) while taking care to maintain the double stranded structure (Figure 2F). 112–117m was taken as a control mutant as it does not contain any predicted nucleotides. In competition UV cross-linking experiment of these IRES mutants with 40S subunits 98–101m mutant showed a moderate reduction in interaction with RPS5 while the interaction of 112–117m mutant with RPS5 was not affected (Figure 2G). *In vitro* translation experiments with 98–101m and 112–117m mutants showed a moderate decrease in IRES activity (Figure 2H).

In addition to using 40S ribosomal subunits as a source for RPS5 in UV cross-linking assays, we also directly used the purified rRPS5. 100 fmol of ^32^P-labelled wild-type RNA and mutant RNAs (Δ61–64, Δ71–73, Δ75–91, ΔSLII) were incubated with increasing concentrations of rRPS5 protein (2 and 4 pmol) in a UV cross-linking assay. Results demonstrated that Δ71–73 and ΔSLII IRES mutants are compromised in interaction with rRPS5 (Figure 2I).

### HCV IRES interaction with RPS5 is required for 80S formation on HCV IRES

**Figure 3.**
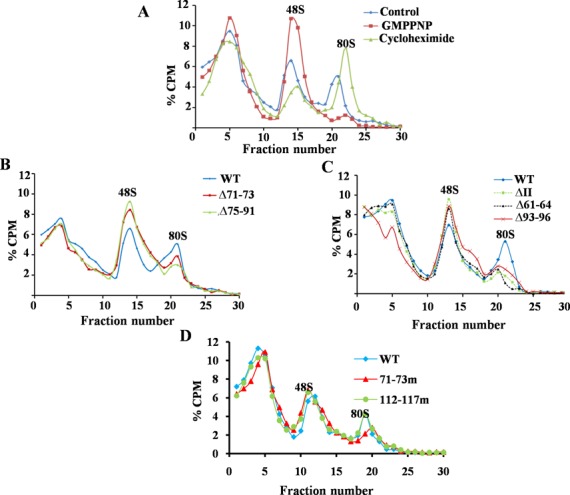
Ribosome assembly on wild type and mutant HCV IRES. (**A**) Ribosome assembly on wild-type ^32^P-labelled HCV IRES RNA in the presence of either cycloheximide or GMPPNP. (**B**–**D**) ^32^P-labelled wild type and mutant HCV IRES RNAs were used as template for translation in RRL, loaded onto 10–30% sucrose gradient and centrifuged to separate the different translation complexes. Different fractions were collected and the radioactivity associated with each fraction was measured. Graph was plotted with % CPM against the respective fraction number.

Next, we examined the effect of the mutations inhibiting HCV IRES–RPS5 interactions on ribosome assembly at the HCV IRES. We confirmed the 48S and 80S peaks in the ribosome assembly profile by using GMPPNP and cycloheximide respectively. As expected GMPPNP and cycloheximide stalled translation at the 48S and 80S stages, respectively (Figure 3A). We performed ribosome assembly experiments with domain II mutants (Δ93–96, Δ61–64, Δ71–73, Δ75–91, 71–73m), which were compromised in their ability to interact with RPS5 and with 112–117m mutant which interacts with RPS5 similar to wild-type HCV IRES (Figure 3B–D). In case of domain II mutants compromised in their ability to interact with RPS5, height and area under the peak corresponding to 80S was reduced while there was an increase in the area and height of the 48S peak, as compared to the wild type. In case of control mutant 112–117m 80S formation was not affected.

### RNA elements in domain IV are critical for IRES–RPS5 interaction

**Figure 4.**
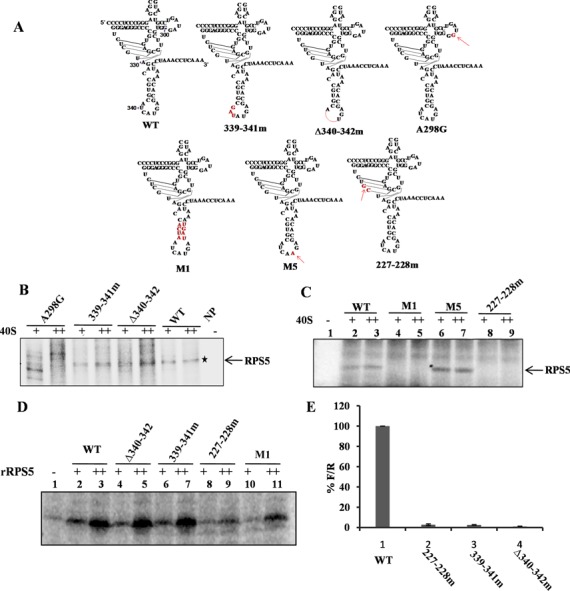
Characterization of RNA elements within HCV IRES domain IV important for HCV IRES–RPS5 interaction. (**A**) Schematic representation of HCV IRES domain IV wild type and mutant constructs used in the study. Mutated regions are shown in red. (**B** and **C**) Direct UV cross-linking of increasing concentrations of 40S ribosomal subunits (5 and 10 pmol) with ^32^P-labelled wild type or domain IV mutant IRES RNAs (100 fmol). This is a representative image of three independent experiments. (**D**) Direct UV cross-linking of increasing concentration of recombinant RPS5 (2 and 4 pmol) with wild-type α-^32^P HCV IRES or domain IV IRES mutants (100 fmol). This is a representative image of three independent experiments. (**E**) *In vitro* translation of wild type and mutant HCV bicistronic RNAs using rabbit reticulocyte lysate (RRL). Graph represents the percentage luciferase activity (% of F/R). Graph represents the average of three independent experiments.

Further, to characterize the interaction between domain IV and RPS5; we generated deletion (Δ340–342) and substitution mutants (339–341m) in domain IV on the basis of computational modelling (Figure 4A). The previously reported A298G mutation (15) was used along with the other two mutants in a direct UV cross-linking assay where the intensity of the UV cross-linked protein is directly correlated with the affinity of the protein for the RNA. In this assay, radiolabelled wild type and mutant IRES RNAs (100 fmol) were incubated with increasing concentration of 40S subunit (5 and 10 pmol), cross-linked by UV irradiation and resolved on a SDS-PAGE. In these studies Δ340–342 and A298G mutants showed reduced cross-linking with RPS5 while 339–341m showed only marginal reduction in interaction with RPS5 (Figure 4B). These mutations significantly affected the IRES activity *in vitro* (Figure 4E).

In addition to these mutations, three more substitution mutations were generated in the pseudo knot (227–228m) and domain IV (M1and M5) (Figure 4A) to study the role of pseudo knot and stem region of domain IV in IRES–RPS5 interaction. UV cross-linking with increasing concentrations of 40S ribosomal subunits (5 and 10 pmol) showed no appreciable binding of 227–228m and M1 IRES mutant RNAs with the RPS5, while M5 cross-linking with RPS5 was found to be marginally increased (Figure 4C). Additionally, *in vitro* translation assay using HCV bicistronic RNA containing respective mutations showed that mutation in 227–228m RNA severely affected HCV IRES activity (Figure 4E). In addition to 40S subunits, we performed direct UV cross-linking of recombinant RPS5 with ^32^P labelled wild type and IRES mutant RNAs (339–341m, Δ340–342, 227–228m, M1). Results demonstrated that 227–228m and M1 IRES mutants are compromised in interacting with rRPS5 (Figure 4D).

### Interaction of RPS5 with HCV RNA is unique to HCV IRES

**Figure 5.**
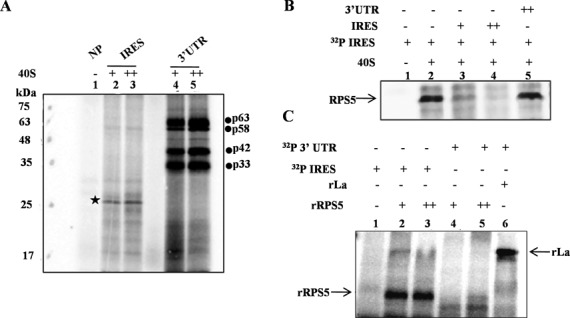
Interaction of RPS5 with HCV IRES and 3′ UTR. (**A**) Direct UV cross-linking of either α-^32^P-labelled HCV IRES or 3′ UTR with increasing concentrations of 40S ribosomal subunit (5 and 10 pmol) This is a representative image of three independent experiments. (**B**) Competition UV cross-linking of α-^32^P-labelled HCV IRES with 40S ribosomal subunit (10pmol) in the presence of either unlabelled HCV IRES RNA (75- and 150-fold) or 3′ UTR RNA (150-fold). This is a representative image of three independent experiments. (**C**) Direct UV cross-linking of α-^32^P-labelled HCV IRES or 3′ UTR with increasing concentrations of recombinant ribosomal protein S5 (4 and 8 pmol). Lane number 6 corresponds to UV cross-linking of La protein with α-^32^P-labelled 3′ UTR as a control for the integrity of the 3′ UTR probe. This is a representative image of three independent experiments.

Recent reports show that in addition to the HCV IRES, 40S ribosome subunit also binds to the 3′ UTR of HCV and affects IRES-mediated translation (21). To characterize the binding of 40S with HCV 3′ UTR, we performed direct UV cross-linking of both ^32^P-labelled HCV IRES and 3′ UTR with increasing concentrations of 40S ribosomal subunits (5 and 10 pmol). Interestingly, RPS5 was found to bind uniquely to the HCV IRES but not the 3′ UTR (Figure 5A). However, we found that HCV 3′ UTR interacts with other ribosomal or ribosome associated proteins of molecular weight ≈35, ≈40, ≈63 and ≈58 kDa. Further, competition UV cross-linking experiments with 75- and 150-fold molar excess of HCV IRES and 3′ UTR unlabelled RNAs demonstrated that RPS5 does not interact with HCV 3′ UTR. Results further confirmed the specificity of interaction of RPS5 with the HCV IRES (Figure 5B). Binding of recombinant RPS5 (2 and 4 pmol) with HCV 3′ UTR (if any) was further investigated by direct UV cross-linking experiment (Figure 5C). Results showed that rRPS5 interacts only with HCV IRES but not with the 3′ UTR supporting the results obtained in the context of 40S subunits. Recombinant La protein was able to bind to HCV 3′ UTR under the same condition, which served as a positive control in the experiment (Figure 5C).

### Partial knockdown of RPS5 inhibits HCV translation

**Figure 6.**
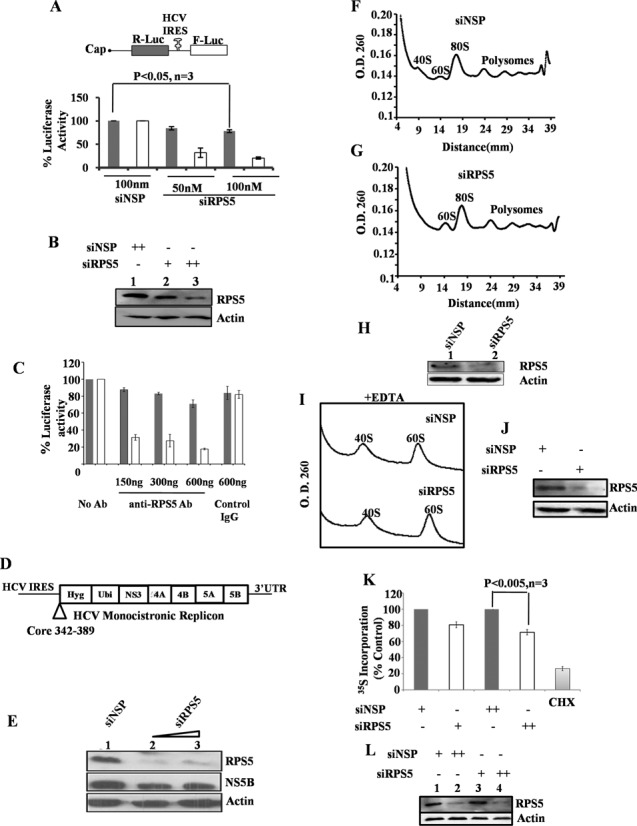
Role of RPS5 in HCV translation and replication. (**A**) Effect of partial knockdown of RPS5 on HCV IRES activity. Huh7 cells were transfected with siNSP RNA or siRPS5 RNA. 48 h later, cells were again transfected with HCV bicistronic RNA. Cells were harvested 10 h post-transfection (5 h after changing serum free media with DMEM plus serum). Graph represents the average of three independent experiments. (**B**) Western blot depicting the partial knockdown of RPS5. (**C**) Inhibition of HCV IRES activity using anti-RPS5 antibody. HCV bicistronic RNA was translated using RRL in the presence of increasing concentration of anti-RPS5 antibody. Non-specific IgG was used as control. (**D**) Schematic of HCV monocistronic replicon RNA. (**E**) Western blot depicting the effect of partial knockdown of RPS5 on HCV protein synthesis. Partial knockdown of RPS5 was achieved by transfection of 50 or 100 nM siRPS5 RNA into Huh7 cells harboring HCV sub genomic replicon (Rep2a). siNSP RNA transfection was used as control. Cells were harvested 72 h post-transfection. (**F** and **G**) Effect of partial knockdown of RPS5 on polysome profile. Huh7 cells were transfected with 100 nM siNSP or siRPS5 RNA. After 96 h, cells were harvested in lysis buffer and lysate was fractionated on a 15–50% sucrose gradient. Polysome profile was obtained by plotting O.D. 260 of each fraction against the distance (mm). (**H**) Western blot indicating the partial knockdown of RPS5 protein. (**I**) Effect of partial knockdown of RPS5 on abundance of 40S and 60S subunits in the cell. Huh7 cells were transfected with 100 nM siNSP or siRPS5 RNA. After 96 h cells were harvested in lysis buffer and lysate was fractionated on a 5–30% sucrose gradient containing 25 mM EDTA. Profile was obtained by plotting O.D. 260 against distance (mm). This is a representative profile of three independent experiments. (**J**) Western blot indicating the partial knockdown of RPS5 protein. (**K**) Effect of partial knockdown of RPS5 on protein synthesis by L-[35S]-methionine incorporation. Cells were transfected with 50 and 100 nM of either siNSP or siRPS5 RNA. After 48 h, ^35^S-methionine incorporation assay was performed and the percentage incorporation was plot. Graph represents the average of three independent experiments. (**L**) Western blot depicting the partial knockdown of RPS5.

Now to study the role of RPS5 on HCV translation, cells were first transfected with siRPS5 (50 and 100 nM) and 48 h post-transfection, cells were again transfected with HCV bicistronic RNA. After 10 h of transfection, cells were harvested and R-Luc and F-Luc activities were measured (Figure 6A and B). siNSP transfected cells were considered as control. In the bicistronic construct, R-Luc activity represents cap-dependent translation and F-Luc activity represents translation mediated by HCV IRES. Partial knockdown of RPS5 preferentially inhibited HCV IRES activity with only marginal effect on cap-dependent translation at higher concentration of siRPS5.To further confirm the role RPS5 in HCV translation we performed *in vitro* translation of bicistronic RNA using RRL system in presence of increasing concentrations of either anti-RPS5 polyclonal antibody (150, 300 and 600 ng) or non-specific antibody (control IgG; Imgenex) (Figure 6C). Addition of increasing concentrations of anti-RPS5 Ab preferentially inhibited HCV IRES activity, further proving the role of RPS5 in HCV translation. However, at higher concentration, anti-RPS5 antibody marginally inhibited cap dependent translation. Further, we extended our study to HCV sub-genomic replicon system. RPS5 was partially silenced using 50nM and 100nM siRPS5 RNA in Huh7 cells harbouring HCV sub-genomic replicon (Rep2a). Cells were lysed 72 h after siRNA knockdown and NS5B and RPS5 levels were determined by Western blot analysis. Partial knockdown of RPS5 resulted in the reduction of HCV-NS5B protein levels in Rep2a cells, suggesting inhibition of translation of HCV RNA due to partial silencing of RPS5 in the context of HCV replicon as well (Figure 6D and E).

Since RPS5 is a ribosomal protein, we checked for the levels of ribosomal subunits and polysomes upon silencing of RPS5. Cells were transfected with 100 nM of siRPS5 and harvested after 96 h in lysis buffer. siNSP RNA was used as control. Lysate equivalent of 200 μg RNA was loaded for each sample on 15–50% sucrose gradient and resolved by ultra-centrifugation. We noticed that knockdown of RPS5 using 100 nM siRPS5 leads to a decrease in the abundance of free 40S subunits without significantly affecting the polysome profile as compared to siNSP (Figure 6F–H)). In accordance with this observation, when the siRPS5 transfected cell lysate was analysed on a 5–30% sucrose gradient in the presence of EDTA, we observed a reduction in the 40S subunit level and hence a reduction in the 40S/60S ratio by 24.61% (Figure 6I and J). To analyse the effect of partial knockdown of RPS5 on global protein synthesis, metabolic labelling (using ^35^S -methionine) experiment was carried out (Figure 6K and L). Huh7 cells were transfected with increasing concentrations of either siRPS5 or siNSP (50 and 100 nM). After 48 h, cells were starved in minus methionine medium for 1 h, followed by labelling with 100 μCi of ^35^S-methionine for 30 min. After metabolic labelling cells were lysed in RIPA buffer and protein was precipitated with ice-cold TCA. Counts were taken using a liquid scintillation counter and % ^35^S-methionine incorporation for each sample was plotted (Figure 6K and L). We observed that cycloheximide treatment (100 μg/ml), used as a positive control, reduced the protein levels by ∼75%. Partial knockdown of RPS5 led to a marginal decrease in the global translation as compared to the siNSP (Figure 6K).

## DISCUSSION

During the initiation of HCV translation, 40S ribosomal subunit binds to uncapped HCV RNA, independent of canonical translation initiation factors. This unique HCV IRES–40S complex formation is mediated by interaction between RNA elements present in HCV IRES and 40S ribosomal proteins. We have studied the interaction of ribosomal protein S5 (RPS5) with the HCV IRES and its possible role in HCV IRES activity. Computational modelling of HCV IRES with 40S ribosome subunit complex suggested that domains II and IV of HCV IRES interact with RPS5 and are in close proximity in the HCV IRES–40S complex. This result is in accordance with the earlier report on tertiary interactions between domains II and IV (34). We have predicted the region of RPS5 and HCV IRES elements involved in HCV IRES–RPS5 interaction and further validated them by mutational studies.

S5M1 peptide derived from RPS5 interacted specifically with HCV IRES while control peptide NSPS5 derived from N terminus of RPS5 containing charged amino acids comparable with those in S5M1 failed to interact. This suggests that the unique sequence and structure of S5M1 are responsible for its interaction with HCV IRES. S5C2 peptide derived from the C-terminus of RPS5 did not show significant interaction with IRES, even though it contained computationally predicted IRES interacting amino acids probably because these amino acids contribute to IRES–RPS5 interaction only in the context of full length protein but not in isolation as a peptide. Further, S5M1 peptide contains a sequence which corresponds to a beta hairpin structure in RPS5. Beta hairpin structures are known to be critical for RNA–protein interactions. For example, the beta hairpin structure present in Tat protein has been reported to play an important role in its interaction with TAR RNA (35). Further, we observed that deletion of the beta hairpin structure in RPS5 protein completely abrogated its interaction with HCV IRES. Together, these results prove that beta hairpin structure present in RPS5 is critical for its interaction with HCV IRES.

In the context of the HCV IRES, predicted contact points in domains II and IV were studied with respect to their interaction with RPS5 and HCV IRES activity. Interestingly, we observed that deletion of RNA elements (Δ75–91,Δ71–73, Δ61–64 and Δ93–96) affected IRES–RPS5 interaction more severely compared to the respective substitution mutations in the context of 40S subunit (83–86m,71–73m, 61–64m and 93–96m) suggesting that structural integrity of domain II is more critical than the sequence of RNA elements. These results are in accordance with the earlier report (15). In contrast to these observations with domain II, in case of domain IV, substitution mutants M1 and 227–228m showed drastically reduced binding with RPS5 suggesting that sequence of domain IV region is critical for its interaction with RPS5.

Apart from the RNA elements predicted to interact with RPS5, few mutations (Δ61–64, Δ93–96 & 227–228m) present in the neighbouring region also severely affected IRES interaction with RPS5 suggesting that they are also important for the interaction. In 227–228m mutant, pseudo knot integrity was affected which in turn might have affected the proper positioning of domain IV in the IRES–40S complex and thus reduced the interaction with RPS5. As pseudo knot integrity is necessary for proper positioning of 40S at initiator AUG (36) it is possible that RPS5 interaction with HCV IRES might play a role in proper positioning of 40S subunit. Interestingly, direct UV cross-linking studies demonstrated that Δ71–73, ΔSLII, 227–228m and M1 mutants interact poorly with recombinant RPS5 while Δ340–342, 339–341m, Δ61–64 & Δ75–91 mutants show interaction similar to the wild type. These results suggest that 71–73 region of domain II, the pseudoknot and GCAC motif present in domain IV are directly involved in IRES–RPS5 interaction. Probably, the Δ61–64 & Δ75–91mutants change the structure and position of domain II on the 40S subunit and hence indirectly affect the IRES-RPS5 interaction. Mutations in either domain II or domain IV severely affect the full-length IRES interaction with RPS5. Hence, we hypothesise that binding of the beta hairpin structure in RPS5 to domain II could bring domain IV in close proximity, thus allowing domain IV also to interact with RPS5 in the IRES–40S complex. Mutations in domain II could affect proper positioning of the ORF into the mRNA binding channel (37) and consequently the interaction of domain IV with RPS5. However, the effect of mutations in domain II on the structure of domain IV in HCV IRES cannot be ruled out. Mutations in domain IV could affect the structure or relative position of domain II in the HCV IRES–40S complex which might subsequently affect the interaction of domain II with RPS5 which may in turn affect the interaction domain IV with RPS5. So we believe that cross talk between domain II and domain IV involving beta hairpin structure present in the RPS5 affects IRES–RPS5 interaction to regulate HCV translation. As reported earlier (38), this cross talk might induce a conformational transition of the 18S rRNA in the region of nucleotide G1639, which enables initiator tRNA to be accepted at the ribosomal P site and subsequently formation of functional 48S complex.

All the mutations in HCV IRES affecting the interaction with RPS5 also severely affected HCV IRES activity proving the importance of HCV IRES and RPS5 interaction in IRES activity. Effect of some of the domain II mutations reported in the present study on HCV IRES activity is in accordance with earlier report (39). However, few mutations which did not affect interaction of IRES with RPS5, also moderately affected the IRES activity suggesting that mutated regions might have affected interaction of IRES with other ribosomal proteins. Formation of 80S was inhibited in the case of Δ75–91, Δ71–73, Δ93–96, Δ61–64 and 71–73m mutants, which were also compromised in binding to RPS5 while 48S formation was slightly increased. In case of the 112–117m IRES mutant, which binds to RPS5 as well as the wild type, 48S and 80S formation on the HCV IRES were not affected. As reported in earlier studies, apical loop of domain II plays an important role in HCV IRES activity (33). We do not rule out the possibility that mutations in the region of domain II that are predicted to interact with RPS5 might affect the positioning of the apical loop of domain II and hence inhibit HCV IRES activity. However, in our ribosome assembly experiments, we have observed a decrease in 80S formation on the IRES whereas apical loop of domain II has been reported to regulate the first ribosomal translocation event without affecting the 80S formation on HCV IRES (33). This indicates that although these mutations might affect the positioning of the apical loop, they affect 80S assembly through a mechanism that is independent of the apical loop involvement and thus IRES activity. We have also used both substitution and deletion mutations in most of our experiments. 71–73m is a substitution mutation in the loop region, which is unlikely to affect the structure of domain II and its relative position on the ribosome. But it still affected the IRES–RPS5 interaction and 80S formation. In addition, we have performed ribosome assembly with the 112–117m which can still cross-link with RPS5 and we observed that it could not inhibit 80S complex formation. This further highlights the importance of IRES–RPS5 interaction in 80S formation.These observations indicate that domain II interaction with RPS5 is necessary for the formation of 80S from the 48S stage. From cryo-EM studies people have shown that deletion of domain II affects the change in 40S conformation that occurs upon binding to IRES (7) and thus affects 80S formation (8). So, we hypothesize that domain II-RPS5 interaction is important in the proper conformational change of 40S and thus for the formation of 80S. This hypothesis explains the inhibition of 80S formation in case of Δ75–91, Δ71–73, Δ93–96, Δ61–64, 71–73m mutations in domain II.

In our assay system, using recombinant RPS5 and 40S subunit, RPS5 showed interaction with HCV IRES but not with 3′ UTR. So the reported interaction of 40S ribosome subunit with the 3′ UTR might involve proteins other than RPS5 present in the exit site of ribosome. Alternatively, the fact that the 3′UTR does not interact with RPS5 could be because of its relative position in the 40S subunit or because the RNA binding regions in RPS5 do not recognize the 3′UTR.

We observed that partial knockdown of RPS5 inhibited HCV IRES mediated translation *ex vivo*. Recently reports have demonstrated that IRES function can be affected by reduction in the free 40S subunit level (31). Thus, the reduction in 40S abundance that we observed in our polysome profile with or without EDTA upon partial knockdown of RPS5 might be responsible for preferential inhibition of the HCV translation. This decrease in free 40S abundance did not affect global translation as much as HCV RNA translation as seen by metabolic labelling. When RPS5 was blocked in 40S ribosome subunits using anti-RPS5 antibody, there was preferential inhibition of HCV IRES activity suggesting a direct role of RPS5 in HCV translation.

Present study sheds light on the RNA binding region of RPS5 and RNA elements of HCV IRES involved in IRES–RPS5 interaction. Beta hairpin present in RPS5 was found to be critical for IRES–RPS5 interaction could retain its interaction with IRES even in isolation as part of a peptide. Domain II–RPS5 interaction was highly dependent on the structural integrity of RNA elements in domain II with moderate contribution by the RNA sequence. In case of domain IV, both the sequence and structure of loop and stem regions were important for IRES–RPS5 interaction. Results with domain II and domain IV mutants suggest an interplay between domains II and IV involving RPS5, which might be critical for functional 48S complex formation. Interaction of domain II and RPS5 was found to be necessary for 80S formation and thus for IRES activity. Also RPS5 was not involved in 3′ UTR–40S interaction while it was important for functional IRES–40S complex formation. siRNA and antibody based experiments suggested that RPS5 is important for HCV protein synthesis *in vitro and ex vivo*. Observations made in the present study provide new insights into RPS5–IRES interaction and its role in translation initiation. This study can also be used as a basis for designing peptide-mimics interfering with RPS5–IRES interaction as a potential antiviral molecule and perform site specific photo cross-linking experiments to further explore RNA–protein interactions involved in HCV IRES–40S complex formation.

## SUPPLEMENTARY DATA

Supplementary Data are available at NAR Online.

SUPPLEMENTARY DATA
