# Supplementary material for: The beta hairpin structure within ribosomal protein S5 mediates interplay between domains II and IV and regulates HCV IRES function
Source: Nucleic Acids Res. 2015 Feb 24;43(5):2888–901. doi: 10.1093/nar/gkv110 (PMC4357715; doi:10.1093/nar/gkv110)
Supplement: SUPPLEMENTARY DATA [file supp_43_5_2888__index.html]

The beta hairpin structure within ribosomal protein S5 mediates interplay between domains II and IV and regulates HCV IRES function — SUPPLEMENTARY DATA 

# The beta hairpin structure within ribosomal protein S5 mediates interplay between domains II and IV and regulates HCV IRES function

## SUPPLEMENTARY DATA

**Files in this Data Supplement:**

- SUPPLEMENTARY DATA
