# Supplementary material for: The beta hairpin structure within ribosomal protein S5 mediates interplay between domains II and IV and regulates HCV IRES function
Source: Nucleic Acids Res. 2015 Feb 24;43(5):2888–901. doi: 10.1093/nar/gkv110 (PMC4357715; doi:10.1093/nar/gkv110)
Supplement: SUPPLEMENTARY DATA [file supp_gkv110_nar-03070-r-2014-File002.pdf]

Supplementary Figure S1

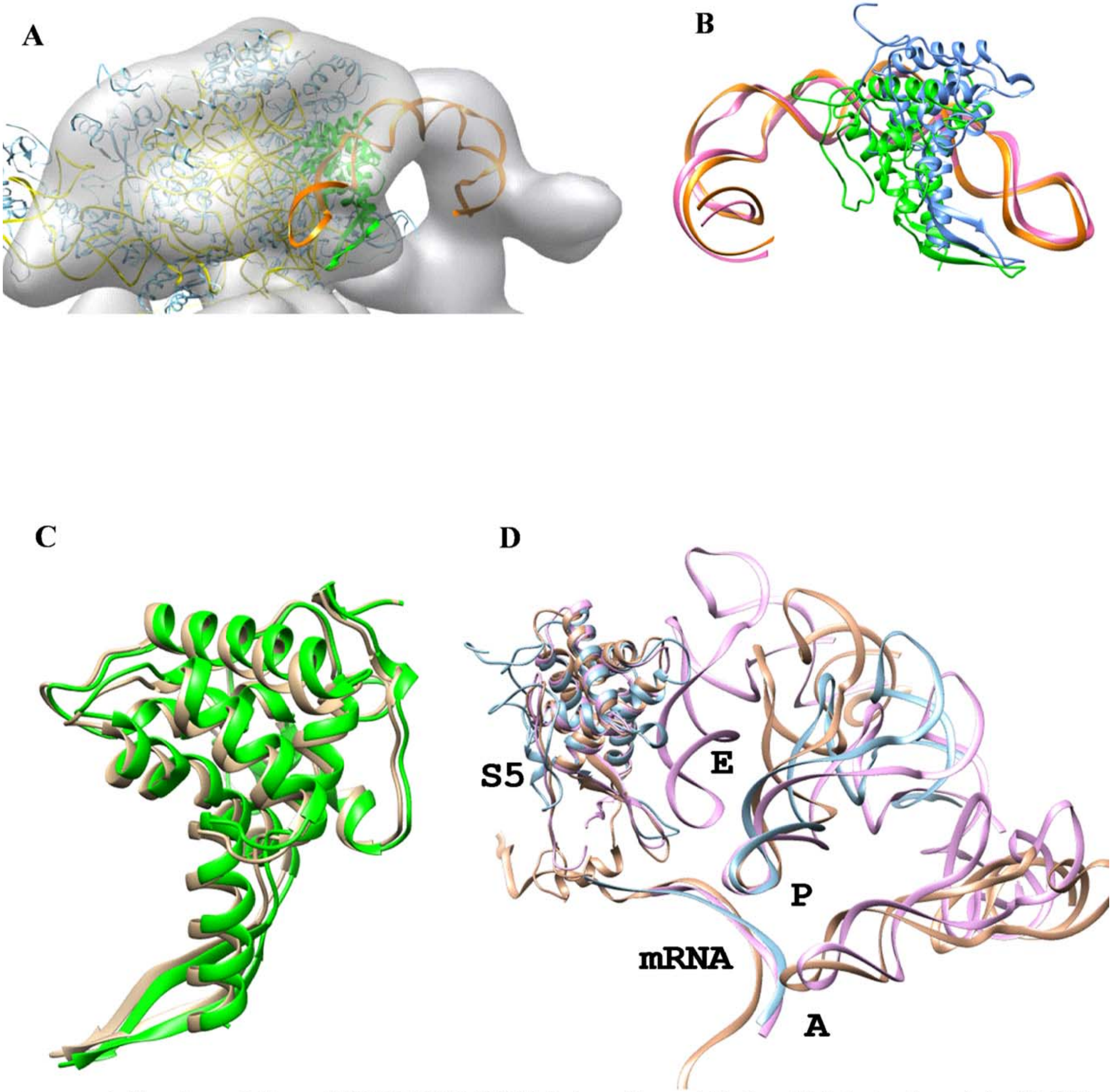

Computational modeling of HCV IRES-RPS5 interactions. (A) CryoEM derived model of RPS5 and domain II interaction. The figure highlights the Exit site region of 40S at the 80S interface. The relative orientation of RPS5 (green) and domain II (brown) obtained by optimizing the fit of human 40S ribosomal head (rRNA in yellow and proteins in blue) (39) and NMR model of domain II (PDB ID: 1P5O) (17), is shown. **(B)** Comparison of the cryoEM derived model (domain II in magenta and RPS5 in blue) with the docking based model (domain II in brown and RPS5 in green). **(C)** Comparison of S5 models obtained by fitting crystal structure of Tetrahymena 40S ribosomal subunit (orange) (45) and human 40S subunit (green) (39), in the IRES-80S complex cryoEM density (16). **(D)** Structural interactions between ribosomal protein S5 (prokaryotic S7), mRNA and E (magenta) site tRNA. The figure shows the superposition of ribosomal protein S7 from two crystals structures with PDB IDs 3J10 (20) (orange) and 2WDM (21) (magenta) and 4KZZ (44) (blue). The associated mRNA and tRNA coordinates (A, P and E site) are shown.

Supplementary Table 1.

Aminoacid:Nucleotide	Aminoacid:Nucleotide	Aminoacid:Nucleotide
R130: U340	R164: U91	Y188: A72,A73
A131: C341	N165: G71	K191: A73
T133: G87	I166: A70	K192: A73
V134: U86	K167: A70	R198: A74
R136: C84	K182: A99	
L103: G100	N186: U97	

List of amino acids of RPS5 and nucleotides of HCV IRES involved in HCV IRES-RPS5 interaction.
